# The effects of resistance training with or without peanut protein supplementation on skeletal muscle and strength adaptations in older individuals

**DOI:** 10.1186/s12970-020-00397-y

**Published:** 2020-12-14

**Authors:** Donald A. Lamb, Johnathon H. Moore, Morgan A. Smith, Christopher G. Vann, Shelby C. Osburn, Bradley A. Ruple, Carlton D. Fox, Kristen S. Smith, Olivia M. Altonji, Zade M. Power, Annsley E. Cerovsky, C. Owen Ross, Andy T. Cao, Michael D. Goodlett, Kevin W. Huggins, Andrew D. Fruge, Kaelin C. Young, Michael D. Roberts

**Affiliations:** 1 Department of Nutrition, Dietetics and Hospitality Management, Auburn University, Auburn, AL 36849 USA; 2 Molecular and Applied Sciences Laboratory, Applied Physiology Laboratory, School of Kinesiology, Auburn University, 301 Wire Road, Office 260, Auburn, AL 36849 USA; 3 Athletics Department, Auburn University, Auburn, AL 36849 USA; 4 Edward Via College of Osteopathic Medicine Auburn, Auburn, AL 36832 USA

**Keywords:** Muscle, Resistance training, Aging, Peanut protein supplementation

## Abstract

Several studies suggest resistance training (RT) while supplementing with various protein supplements can enhance strength and muscle mass in older individuals. However, to date, no study has examined the effects of RT with a peanut protein powder (PP) supplement on these outcomes. Herein, 39 older, untrained individuals (*n* = 17 female, *n* = 22 male; age = 58.6 ± 8.0 years; body mass index =28.7 ± 5.8) completed a 6-week (*n* = 22) or 10-week (*n* = 17) RT program, where full-body training was implemented twice weekly (ClinicalTrials.gov trial registration NCT04015479; registered July 11, 2019). Participants in each program were randomly assigned to consume either a PP supplement once per day (75 total g powder providing 30 g protein, > 9.2 g essential amino acids, ~ 315 kcal; *n* = 20) or no supplement (CTL; *n* = 19). Right leg vastus lateralis (VL) muscle biopsies were obtained prior to and 24 h following the first training bout in all participants to assess the change in myofibrillar protein synthetic rates (MyoPS) as measured via the deuterium-oxide (D_2_O) tracer method. Pre- and Post-intervention testing in all participants was conducted using dual energy x-ray absorptiometry (DXA), VL ultrasound imaging, a peripheral quantitative computed tomography (pQCT) scan at the mid-thigh, and right leg isokinetic dynamometer assessments. Integrated MyoPS rates over a 24-h period were not significantly different (*p* < 0.05) between supplement groups following the first training bout. Regarding chronic changes, there were no significant supplement-by-time interactions in DXA-derived fat mass, lean soft tissue mass or percent body fat between supplementation groups. There was, however, a significant increase in VL thickness in PP versus CTL participants when the 6- and 10-week cohorts were pooled (interaction *p* = 0.041). There was also a significant increase in knee flexion torque in the 10-week PP group versus the CTL group (interaction *p* = 0.032). In conclusion, a higher-protein, defatted peanut powder supplement in combination with RT positively affects select markers of muscle hypertrophy and strength in an untrained, older adult population. Moreover, subanalyses indicated that gender did not play a role in these adaptations.

## Introduction

The gradual age-related decrease in muscle mass and strength, termed sarcopenia, culminates in a reduction of nearly 40% of an individual’s total muscle mass by the eighth decade of life [1–3]. Sarcopenia coincides with cellular, neuromuscular and metabolic perturbations [4]. Not only does sarcopenia directly contribute to increased frailty and fragility [5, 6], but it indirectly contributes to more serious health consequences such as decreased quality of life and premature death [7]. Therefore, interventions that aim to increase muscle mass in older individuals in order to prevent the development of sarcopenia have garnered much attention.

A plethora of research has demonstrated that 8–16 weeks of resistance training (RT) can increase muscle mass and strength in older individuals (reviewed in [8, 9]). Given that protein feeding stimulates an anabolic response in skeletal muscle [10], it stands to reason that combining protein supplementation with RT likely optimizes increases in muscle mass. Animal-based protein sources possess the full complement of essential amino acids needed to stimulate the muscle-building process at the molecular level (i.e., increases in post-meal myofibrillar protein synthesis or MyoPS rates) [11]. Moreover, it has been well documented that dairy-derived protein supplements (e.g., milk or whey protein concentrates or isolates) can enhance increases in muscle mass with RT relative to other protein sources [12]. However, there has been a growing interest in the health benefits of plant-based foods as well as concerns related to the sustainability of procuring animal-based proteins [13]. In this regard, data from the National Health and Nutrition Examination Survey indicate that intakes of plant proteins increased significantly from 1999 to 2010 [14], and there is sentiment that consumers will continue to increase plant protein intake for the foreseeable future [15].

Protein isolates from several plant-based foods (i.e. soy, pea, rice, and hemp) are currently sold to consumers with the intent of supporting the rigorous demands of exercise training. There has also been a recent growth in the popularity and availability of peanut flour and defatted peanut powder. With the exception of containing low methionine and threonine levels, peanut protein possesses a full complement of essential and non-essential amino acids [16]. Relative to other plant-based proteins (e.g., wheat or legumes), peanut protein possesses a relatively high protein digestibility corrected amino acid score (0.70/1.00) [16]. In fact, it has been posited that peanut protein can be used as an ingredient for protein fortification in low-protein food sources [17]. Despite these positive statistics surrounding peanut protein, no study to date has examined if a peanut powder (PP) supplement combined with RT can enhance training adaptations. Therefore, the purpose of this study was two-fold. First, we sought to determine if post-exercise PP supplementation could enhance the MyoPS response to one resistance exercise bout in older participants with no prior formal resistance training experience. Second, we sought to determine if PP supplementation with 10 weeks of RT could enhance muscle quality, body composition and strength in these same participants.

## Methods

### Ethical approval and participant screening

Prior to any data collection, this study was approved by the Auburn University Institutional Review Board (IRB) (Protocol # 19-249 MR 1907), conformed to standards set by the latest revision of the Declaration of Helsinki, and was registered as a clinical trial (NCT04015479). Men and women aged 50–80 years with minimal RT experience, defined here as not having performed structured RT for at least 3 months prior, were recruited for this study. Participants were recruited via flyer, email inquiry and newspaper advertisement. Interested participants were informed of the study and testing procedures either over the phone or face-to-face at the Auburn University School of Kinesiology. Eligibility criteria indicated that potential participants had to: 1) be between the ages of 50–80 years old, 2) not actively be participating in structured RT for at least 3 months prior, 3) be free of metal implants, and 4) possess blood pressure readings within normal ranges, with or without medication (i.e. < 140/90 SBP/DBP). Exclusion criteria included: 1) individuals having a known peanut allergy, 2) individuals having a body mass index ≥35 kg/m^2^, 3) individuals being exposed to medically-necessary radiation in the last 6 months, or 4) individuals having a medical condition contradicting participation in a RT program, giving blood or donating a skeletal muscle biopsy (i.e. blood clotting disorders or taking blood thinning medications). Participants deemed eligible based on the aforementioned criteria provided written and verbal consent to participate. A medical history questionnaire was obtained at the time of consenting and participants were scheduled to return to the Auburn University School of Kinesiology to complete study procedures described below*.*

### Study design

**Fig. 1:**
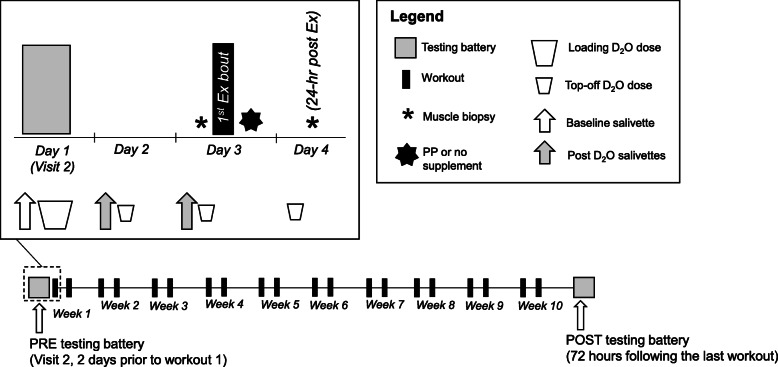
Study Design. Legend: The figure above outlines the study design for the 10-week cohort. Note, that the second (6-week) cohort followed the same experimental procedures with the exception of training duration, which lasted only 6 weeks (or 12 total workouts)

Our original intent was to recruit two separate ten-week cohorts. Due to the SARS-CoV-2 pandemic, we voluntarily decided to end the second cohort after only 6 weeks of training. As such, the primary difference between cohorts was the length of the intervention. The study design for the 10-week and 6-week cohorts is presented in Fig. 1 below.

Briefly, participants in the 10-week cohort reported to the Auburn University School of Kinesiology on 24 separate occasions, whereas participants in the 6-week cohort reported on 16 separate occasions. Visit one (V1) included screening to determine eligibility, gathering consent and obtaining a health history. Visit two (V2; PRE) occurred at least 3 days prior to visit 3 (V3) and included a battery of assessments comprised of urine specific gravity (USG), height and body mass, ultrasound of the right leg vastus lateralis (VL), full body dual energy x-ray absorptiometry (DXA), peripheral quantitative computed tomography (pQCT) scan at the mid-thigh of the right leg and right leg strength assessment using an isokinetic dynamometer. Following the battery of assessments, participants were provided with deuterium oxide (D_2_O)-enriched water, a three-day food log, and three separate salivettes to measure D_2_O enrichment. The food log was returned prior to V3 at each participant’s convenience.

V3 included the participant’s first muscle tissue sample collection, randomization to either the peanut protein supplement group (PP) or wait-list control (CTL), the participant’s first resistance exercise bout, and immediate post-exercise PP supplementation or no supplementation. A complete nutritional breakdown for the PP supplement is presented in Table 1. V4 included the participants’ second muscle tissue sample collection and salivette return. Visit five (V5) through visit 23 (V23) for the 10-week cohort and V5 through visit 15 (V15) for the 6-week cohort included a single RT session. During V23 for the 10-week cohort and V15 for the 6-week cohort participants were provided with their second set of food logs. Visit 24 (V24; POST) for the 10-week cohort and visit 16 (V16; POST) for the 6-week cohort occurred roughly 72 h following V23 and V15, respectively, and included a repeat of the V2 testing battery. Specific testing methodologies are detailed below.

**Table 1:** Amino acid content in PP per serving

Variable	Amount per daily serving (3 scoops or 75 g powder)
*Total protein*^*1*^	30.1 g
*Essential*^*2*^	
Histidine	0.72 g
Isoleucine	1.14 g
Leucine	2.17 g
Lysine	0.87 g
Methionine	0.41 g
Phenylalanine	1.66 g
Threonine	0.86 g
Tryptophan	ND
Valine	1.37 g
*Total essential*	9.2 g
*Non-essential*	
Alanine	1.37 g
Arginine	3.71 g
Asparagine	3.77 g
Cysteine	0.45 g
Glutamic acid	6.26 g
Glycine	1.90 g
Proline	1.37 g
Serine	1.49 g
Tyrosine	1.35 g
*Total non-essential*	21.7 g

These results are from third party testing as described in the methods. Superscript numbers: 1, indicates this was determined by the Dumas method; 2, determined per Eurofin’s Agilent Application note 5990-4547 (2010)

*ND* not determined using the Agilent method

### Pre- and post-intervention testing battery

The testing sessions described below occurred during morning hours (05:00–09:00) following an overnight fast for all but 7 participants who reported to the laboratory after working hours at 17:00–18:30 following a ~ 4–5 h fast.

#### Body composition assessments

During V2 and V24 (10-week participants) or V2 and V16 (6-week participants), participants reported to the Auburn University School of Kinesiology wearing casual sports attire (i.e. athletic shirt and shorts, tennis shoes). Participants submitted a urine sample (~ 5 mL) to assess USG levels using a handheld refractometer (ATAGO; Bellevue, WA, USA). Notably, all participants possessed USG values less than 1.020 indicating that they were well hydrated. Height and body mass were assessed using a digital column scale (Seca 769; Hanover, MD, USA) with mass and height being collected to the nearest 0.1 kg and 0.5 cm, respectively. Thereafter, right leg VL images were captured in the transverse plane using real-time B-mode ultrasonography (LOGIQ S7 Expert, GE Healthcare, USA) utilizing a multi-frequency linear-array transducer (3–12 MHz, GE Healthcare, USA) and subsequently analyzed for VL thickness. Participants were instructed to stand and displace bodyweight to the left leg to ensure the right leg was relaxed. Measurements were standardized by placing the transducer at the midway point between the inguinal crease and proximal border of the patella. All images were captured and analyzed by the same investigator (S.C.O.) with a 24-h test-retest reliability using intraclass correlation coefficient (ICC_3,1_), standard error of the measure (SEM), and minimal difference (MD) to be considered real of 0.991, 0.06, and 0.16 cm, respectively. Participants then underwent a full body dual-energy x-ray absorptiometry (DXA) scan (Lunar Prodigy; GE Corporation, Fairfield, CT, USA) for determination of total lean soft tissue mass (LSTM) and fat mass (FM). Quality assurance testing and calibration were performed the morning of data-collection days to ensure the scanner was operating to manufacturer specification. Scans were analyzed by the same technician using the manufacturer’s standardized software. Test-retest reliability using ICC_3,1_, SEM, and MD were previously determined for LSTM (0.99, 0.36, and 0.99 kg, respectively) and FM (0.99. 0.43, and 1.19 kg). Following the DXA scan, a cross-sectional image of the right thigh at 50% of the femur length was acquired using a pQCT scanner (Stratec XCT 3000, Stratec Medical, Pforzheim, Germany). Scans were acquired using a single 2.4 mm slice thickness, a voxel size of 0.4 mm and scanning speed of 20 mm/sec. All images were analyzed for total muscle cross-sectional area (mCSA, cm^2^) and density (mg/cm^3^) using the pQCT BoneJ plugin freely available through ImageJ analysis software (NIH, Bethesda, MD). All scans were performed and analyzed by the same investigator (K.C.Y.). Test-retest reliability using ICC_3,1_, SEM, and MD was previously determined for mCSA (0.99, 0.84, and 2.32 cm^2^, respectively).

#### Right leg isokinetic strength assessment

Participants performed maximal isokinetic right leg extensions on an isokinetic dynamometer (System 4 Pro, BioDex Medical Systems, Shirley, NY, USA). Participants were fastened to the dynamometer so that the right knee was aligned with the axis of the dynamometer. Seat height was adjusted to ensure the hip angle was approximately 90°. Prior to peak torque assessment, each participant performed a warmup consisting of submaximal to maximal isokinetic knee extensions. Participants then completed five maximal voluntary isokinetic knee extension actions at 60°/sec and 120°/sec. Sets were separated by 60 s of rest. Participants were provided verbal encouragement during each set. The isokinetic extension resulting in the greatest peak torque value was used for analyses. Right leg extensor peak torque testing occurred ~ 1–3 days prior to the muscle biopsy at the PRE (V2) time point in both the 10-week and 6-week cohorts, whereas this test occurred approximately 10 min following the biopsy at the POST time point for the 10-week cohort only (V24). This difference in methodology between time points was due to logistical constraints. However, we have unpublished data suggesting peak torque values are not affected by muscle biopsies when isokinetic testing occurs within a 10-min post-biopsy window [18].

### Supplement randomization and resistance training

During V3, immediately following collection of the first muscle sample, participants were randomized to either consume PP during the intervention (*n* = 20) or after the intervention (*n* = 19). The PP supplement (PBfit; BetterBody Foods, Lindon, UT, USA) claimed to have provided the following per daily serving: 315 kcal, 35 g protein, 10.7 g essential amino acids (where 2.44 g was L-leucine), 9.0 g fat and 22.5 g carbohydrate (with 14.8 g fiber and 7.7 g sugars). Servings were in the form of protein powder provided to participants, and participants were instructed to mix the powder contents (3 full scoops exactly, 75 g) with 16 fluid ounces of tap water prior to drinking. Our research team decided to compare PP supplementation to no supplementation given that this carried more “real world” relevance (i.e., people choose to supplement with protein powder, or nothing at all). We also sent the supplement out for third-party testing (Eurofins; Tucker, GA, USA) to determine total protein and total amino acid content of the supplement. This information can be found in Table 1 below.

Randomization was stratified by gender in blocks of four, hence the slight differences in allocation to group. Afterwards, participants were escorted to the Auburn University School of Kinesiology Fitness and Performance Optimization Laboratory for their first resistance exercise session. Participants were provided detailed instructions on proper posture, technique, range-of-motion, body positioning and breathing to ensure safety. Participants completed supervised RT twice weekly for either 10 weeks or 6 weeks. All RT sessions were separated by at least 48 h to allow for a period of recovery. Each RT session consisted of five exercises including seated leg press, leg extensions, lying leg curls, barbell bench press and cable pull-downs. For each exercise, participants performed three sets of 10–12 repetitions with 1 min of rest between sets. At the end of each set, participants were asked to rate the level of difficulty where 0 = easy, 5 = moderate difficulty and 10 = hard. If values were below 7, weight was modestly added to increase exertion on the subsequent set. If values were 10, or the participant could not complete the set, weight was removed prior to the next set. Participants were encouraged to be as truthful as possible when assessing difficulty and were provided verbal encouragement and feedback during and following each set. The intent of this training method was to consistently challenge participants so that perceived exertion after each set of 10–12 repetitions was at a 7–9 rating. Training data for each participant were logged, allowing us to ensure that training effort was maximized within each training session, and participants were successfully implementing progressive overload in an individualized fashion.

Notably, study personnel supervised all training throughout the study. Participants in the PP group were instructed to consume one daily serving of the PP supplement. On workout days, PP supplements were provided to participants in the PP group immediately following exercise, and supplementation compliance was supervised. On non-workout days, participants were instructed to consume one serving between meals. Product bottles were returned to the study coordinator to ensure compliance to the supplementation protocol.

### Muscle sample collection and integrated myofibrillar protein synthesis rate determination using deuterium oxide

MyoPS rates were determined after the first bout of training with or without PP supplementation using the integrated D_2_O technique. Briefly, participants consumed a total 4.5 mL∙kg^− 1^ of lean body mass (LBM) of D_2_O-enriched water (70 atom percent; Sigma-Aldrich, St. Louis, MO) over the course of four separate days beginning 2 days prior to V2 through V3. Participants were provided with six individual servings of D_2_O. Three of these servings contained 1 mL∙kg^− 1^ LBM D_2_O and were consumed in a single day as a loading phase, and three of these servings contained 0.5 mL∙kg^− 1^ LBM D_2_O and were consumed over the next three consecutive days.

Saliva samples were taken according to the schematic in Fig. 1 in order to estimate whole-body deuterium enrichment prior to and following D_2_O consumption. Briefly, participants were given sterile salivette kits, which contained a cotton swab and two-compartment cryotube (SARSTEDT AG &Co, Nümbrect, Germany). Participants were instructed to: a) chew on the cotton swab for 1 min, b) place the swab back into the top compartment of the tube, and c) place the tubes in their home freezers until they were capable of bringing them directly to the laboratory. Once samples were brought into the laboratory they were stored at − 20 °C. At the end of the study, all salivette tubes were centrifuged for 2 min at 1000 *g* (2 °C). The flow-through saliva was then frozen at − 20 °C until shipment to Metabolic Solutions (Nashua, NH, USA) on dry ice and processed as described below.

Skeletal muscle biopsies at V3 and V4 were obtained from the right thigh (i.e VL; in the same plane as ultrasound and pQCT assessments) midway between the patella and iliac crest using a 5-gauge needle with suction and sterile laboratory procedures. Briefly, upon arrival to the laboratory, participants were instructed to lie in a supine position on an athletic training table. Approximately 5 min afterwards, 1.5 mL of 1% lidocaine was injected subcutaneously above the skeletal muscle fascia, and a small pilot incision was made for needle insertion using a sterile Surgical Blade No. 11 (AD Surgical; Sunnyvale, CA, USA). After 5 min of allowing the anesthetic to take effect, the biopsy needle was inserted into the pilot incision just beyond the fascia and approximately 50–100 mg of skeletal muscle was removed using a double chop method and applied suction [19]. Following biopsies, tissue was rapidly teased of blood and connective tissue and subsequently stored at − 80 °C until the isolation of myofibrils. The day of myofibril isolation, all samples were batch-processed using the recently published MIST method; for further details refer to our recent publication [20]. Thereafter, isolated myofibrils were shipped on dry ice to Metabolic Solutions for tracer analyses as described below.

Saliva samples were analyzed for deuterium enrichment by cavity ring-down spectroscopy using a Liquid Water Isotope Analyzer with automated injection system, version 2 upgrade (Los Gatos Research, Mountain View, CA, USA). Samples were vortexed and spun at 8000 rpm to remove any particulates. The water phase of saliva was injected 6 times, and the average of the last three measurements was used for data analysis. A standard curve was run before and after samples for calculation of deuterium enrichment. Intra-run precision is typically less than 2 delta per mil (parts per thousand) and inter-run precision is typically less than 3.5 delta per mil.

Myofibrillar protein was hydrolyzed for 18 h at 100 °C with 3 ml 6 N HCl. In addition to HCl, 1 mL Dowex H^+^ resin (50Wx8–100; Sigma-Aldrich, Saint Louis, MO, USA) was added to trap released alanine from protein. The amino acids were eluted from the resin using 2 mL of 3 N NH_4_OH. Eluates were evaporated to dryness. The N-acetyl, n-propyl (NAP) derivative of alanine was prepared. The propyl ester is formed by addition of 200 uL propyl acetate and 100 ul BF3:Propanol (14%). Samples were heated at 110 °C for 30 min. Solutions were evaporated to dryness under N_2_ gas at 60 °C. The N-acetyl group was formed by adding 100 uL of 0.1 M diethylamine (DEA) in hexane and 100ul of acetic anhydride and reacted for 20 min at 60 °C. Reagents were dried down with N_2_ gas and low heat. Samples were reconstituted in 100 uL ethyl acetate and placed into an autosampler vial. Myofibrillar preparations were analyzed for deuterated-alanine with a Thermo Finnigan Delta V IRMS coupled to a Thermo Trace GC Ultra with a GC combustion interface III and Conflow IV. The N-acetyl-n-Propyl ester of alanine was analyzed using a splitless injection with CTC Pal autosampler (1 μL), at an injection temperature of 250 °C, and using a Zebron ZB-5 column of 30 m × 0.25 mm × 0.50 μm film thickness (Phenomenex, Torrance, CA, USA). The GC oven was programmed with an initial column temperature of 80 °C with a 2-min hold, followed by a ramp of 30 °C per minute to 330 °C. Compounds eluting off the column were directed into the pyrolysis reactor, heated at 1450 °C and converted to hydrogen gas. The deuterated enrichment was first initially expressed in delta values compared to a calibrated hydrogen gas and then converted to atom % D by standard equations. Methylpalmitate, obtained from Dr. Arndt Schimmelmann (Biogeochemical Laboratories, Indiana University) was used as the calibration standard for the reference hydrogen gas. Intra-run precision for alanine measurements is typically less than 2 delta per mil (parts per thousand) and inter-run precision is typically less than 3 delta per mil.

MyoPS rates over the 24-h period following the first training bout were calculated similar to Bell et al. [21] (see equation below).

$$ FSR\ \left(\%{day}^{-1}\right)=\left[\frac{\left({E}_{Ala2}-{E}_{Ala1}\right)}{E_{BW}\times t}\right]\times 3.7\times 100 $$

Briefly, E_Ala1_ and E_Ala2_ represent ^2^H enrichment in the first and second muscle biopsies, respectively (in atom percent excess). E_BW_ is the average ^2^H enrichment (in atom percent excess) of total body water from the second and third salivettes after subtracting background values from the baseline salivette. *t* is time in the number of days D_2_O was ingested (which equals 1). The 3.7 coefficient adjusts for average ^2^H atoms that can be bound to alanine, and final values were expressed as % synthesis per day by multiplying values by 100.

### Food log analysis

Participants were instructed to self-report their habitual food intake for three consecutive days and return these food logs at V3 and V24 or V16 (10- and 6-week cohort, respectively). Participants were asked not to change their diet in any way, with the exception of PP participants who were instructed to consume the supplement as described above. Study staff entered each food log into the Automated Self-Administered 24-Hour Dietary Assessment tool (ASA24), which uses the United States Department of Agriculture Food and Nutrient Database for Dietary Studies to provide values for 195 nutrients, nutrient ratios and other food components [22].

### Statistical analysis

All statistical analyses were performed using SPSS v26.0 (IBM Corp, Armonk, NY, USA). Independent samples t-tests were used for MyoPS and training volume comparisons between the PP and CTL groups. For all dependent variables over time, repeated measures two-way (group × time; GxT) ANOVAs were performed. When a significant interaction occurred, LSD post hocs were performed between and within groups to determine the level of significance. With the exception of MyoPS data, all data in figures are presented to show the 6-week cohort individually, 10-week cohort individually, and pooled cohorts (6-week and 10-week) collectively. Group, time and GxT *p*-values are provided for each cohort individually and when pooled. Change scores (or delta scores) in key training variables were also calculated by subtracting PRE values from POST values, and these scores were compared between various groups for subanalyses using independent samples t-tests. Pearson correlations were also performed on select variables. Statistical significance was established as *p* < 0.05, and relevant p-values are depicted in-text or within figures.

## Results

### MyoPS response to the first bout of training with or without post-exercise PP supplementation

**Fig. 2:**
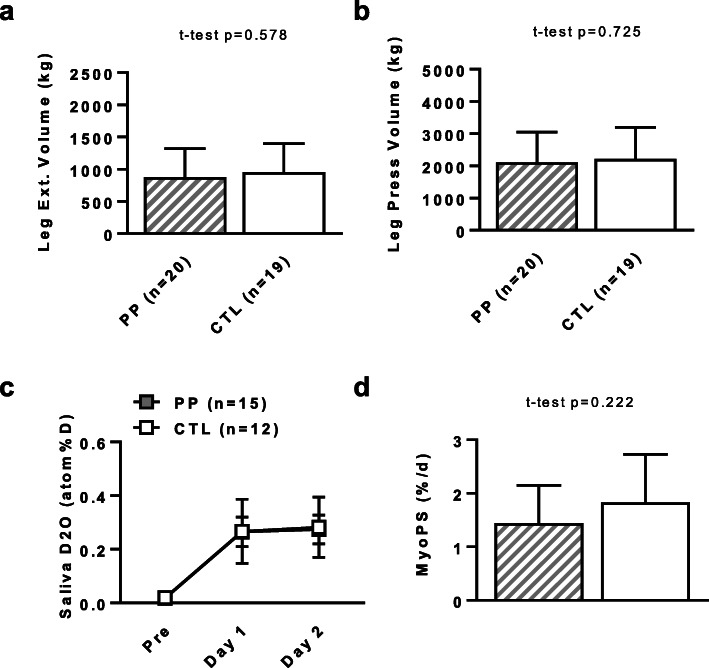
Myofibrillar protein synthesis rates following the first bout of training with or without PP supplementation. Legend: No differences between conditions existed for the leg extensor (panel **a**) or leg press (panel **b**) training volume during the first training bout. Saliva D_2_O enrichment increased from baseline V2 to V3 and V4 regardless of supplementation (panel **c**). Myofibrillar protein synthesis rates 24 h following the first exercise bout did not differ between PP and CTL participants (panel **d**). All data are presented as mean ± standard deviation values. Abbreviations: PP, peanut powder supplemented participants; CTL, non-supplemented participants

Prior to discussing the chronic training adaptations between the PP and CTL groups, the MyoPS responses following the first exercise training bout are first presented. After this portion of the results, the CONSORT diagram, baseline characteristics between groups, chronic training adaptations, and dietary records over the duration of the study will be discussed. There were no significant differences in total leg extension or leg press volumes for the first bout of training between supplementation groups (Fig. 2a and b). Twenty-seven participants (*n* = 15 PP, *N* = 12 CTL) yielded salivettes with viable enrichment values for tracer analysis (Fig. 2c). There was an enrichment effect over time fromV2 to V3 (*p* < 0.001), but no further differences in enrichment from V3 to V4. There was no difference in the 24-h MyoPS rates between supplement groups following the first bout of RT (Fig. 2d).

### Study CONSORT diagram

**Fig. 3:**
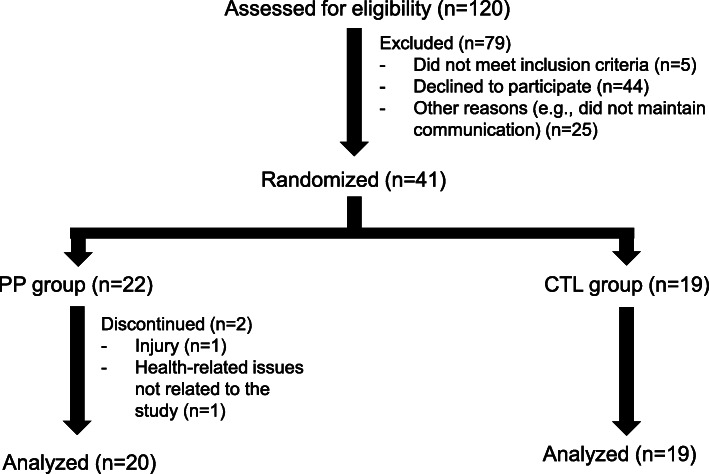
CONSORT Diagram. Legend: The diagram indicates how many individuals were screened and completed the intervention

Figure 3 provides a detailed CONSORT diagram of the study. Briefly, 120 potential participants contacted the study coordinator. Of these, 41 were eligible and agreed to participate in the study, and *n* = 22 were randomized to the PP group whereas *n* = 19 were randomized to the CTL group. Two participants in the PP group had to discontinue the study due to injury from weightlifting (*n* = 1) or health reasons outside of the study (*n* = 1), whereas none of the CTL participants discontinued the study. Thus, *n* = 20 PP participants and *n* = 19 CTL participants were included in most analyses unless stated otherwise in the results, figures, or tables.

### Baseline participant characteristics

Baseline participant characteristics between the PP and CTL cohorts are presented in Table 2. Notably, there were no differences between cohorts regarding body composition metrics. Although there was not a difference between cohorts regarding average age, the age range was rather wide in each group (PP range = 51–79 years old, CTL range = 50–76 years old). Given this limitation, additional statistical analyses considering age stratification were performed key dependent variables later in this section.

**Table 2:** Baseline Participant Characteristics

Variable (units)	Mean ± SD		*p*-value
Gender	PP	12 M / 8 F	N/A
	CTL	10 M / 9 F	
Age (years)	PP	60 ± 9	*p* = 0.61
	CTL	58 ± 7	
Height (cm)	PP	171.7 ± 8.3	*p* = 0.41
	CTL	172.0 ± 9.3	
Weight (kg)	PP	84.9 ± 17.6	*p* = 0.52
	CTL	88.8 ± 20.5	
BMI (kg/m^2^)	PP	27.8 ± 5.5	*p* = 0.23
	CTL	29.7 ± 6.2	
DXA % body fat (%)	PP	36.0 ± 7.1	*p* = 0.92
	CTL	36.2 ± 7.6	

Baseline participant characteristics are presented as means ± standard deviation values

*Abbreviations*: *PP* peanut powder supplemented participants (*n* = 20), *CTL* non-supplemented participants (*n* = 19), *DXA* dual x-ray absorptiometry, *BMI* body mass index, *cm* centimeters, *kg* kilograms, *kg/m*^*2*^ kilograms per meter squared

### Food log data

Data from the pre- and post-training food logs between the PP and CTL cohorts are presented in Table 3. Thirty-one participants (*n* = 16 PP, *n* = 15 CTL) returned completed food logs suitable for analyses, and data from the PP group includes one serving of PP per day. Notably, there was a significant interaction for protein consumption in the pooled participants (*p* = 0.008). While protein consumption decreased slightly in the CTL group (Pre = 71 ± 30 g versus Post = 68 ± 23 g, *p* > 0.05), protein consumption significantly increased in the PP group (Pre = 95 ± 24 g versus Post = 119 ± 22 g, *p* = 0.007). Additionally, fiber consumption significantly increased in both the 10-week (*p* = 0.013) and pooled cohorts (*p* = 0.003). Despite a slight decrease in fiber consumption for the 10-week CTL cohort (Pre = 13 ± 4 g versus Post = 12 ± 7 g, *p* > 0.05), there was a significant increase in fiber consumption for the 10-week PP cohort (Pre = 19 ± 15 g versus Post = 26 ± 9 g, *p* = 0.032). There was also a difference in fiber consumption of the pooled cohorts. While there was no increase in fiber consumption for the CTL cohort (Pre = 14 ± 4 g versus 15 ± 6 g, *p* > 0.05), there was a significant increase in fiber consumption for the PP cohort (Pre = 19 ± 10 g versus Post = 27 ± 6 g, *p* < 0.001). The increase in daily fiber intake in the PP groups was mainly attributed to the ~ 15 g/d of fiber received from the nutritional supplement.

**Table 3:** Pre- and post-intervention food recall data

Variable	Pooled CTL (*n* = 15)		Pooled PP (*n* = 16)	
	PRE	POST	PRE	POST
Energy (kcal)	1799 ± 647	1683 ± 569	2106 ± 383	2181 ± 412
Pro (g)	71 ± 30	68 ± 23	95 ± 24	119 ± 22*#
Fat (g)	74 ± 31	66 ± 27	89 ± 18	91 ± 21
CHO (g)	210 ± 82	206 ± 71	216 ± 75	209 ± 49
Sugar (g)	105 ± 54	92 ± 54	124 ± 63	98 ± 44
Fiber (g)	14 ± 4	15 ± 6	19 ± 10	27 ± 6*#
	10-week CTL (*n* = 7)		10-week PP (*n* = 6)	
Energy (kcal)	1682 ± 737	1523 ± 578	2130 ± 336	2150 ± 456
Pro (g)	72 ± 36	62 ± 19	92 ± 20	111 ± 14#
Fat (g)	74 ± 37	60 ± 25	83 ± 16	85 ± 22
CHO (g)	178 ± 70	180 ± 79	214 ± 75	208 ± 49
Sugar (g)	83 ± 32	85 ± 67	90 ± 55	87 ± 29
Fiber (g)	13 ± 4	12 ± 7	19 ± 15	26 ± 9*#
	6-week CTL (*n* = 8)		6-week PP (*n* = 10)	
Energy (kcal)	1901 ± 588	1823 ± 560	2092 ± 426	2200 ± 408
Pro (g)	71 ± 27	73 ± 27	96 ± 27	123 ± 26
Fat (g)	74 ± 29	71 ± 29	93 ± 19	94 ± 21
CHO (g)	239 ± 84	229 ± 58	218 ± 79	209 ± 51
Sugar (g)	124 ± 63	98 ± 44	87 ± 39	79 ± 31
Fiber (g)	15 ± 5	17 ± 3	19 ± 6	28 ± 5

These data are from the 3-day food recalls averaged to intakes per 1 day. All data are presented as means ± standard deviation values

*Abbreviations*: *PP* peanut powder supplemented participants, *CTL* non-supplemented participants, *kcal* kilocalorie, *g* gram, *Pro* protein, *CHO* carbohydrate

Symbols: *, significant increase within PP from Pre to Post (*p* < 0.05); #, PP > CTL at Post (*p* < 0.05)

### Differences in resistance training volumes after 6- or 10-weeks of training

**Fig. 4:**
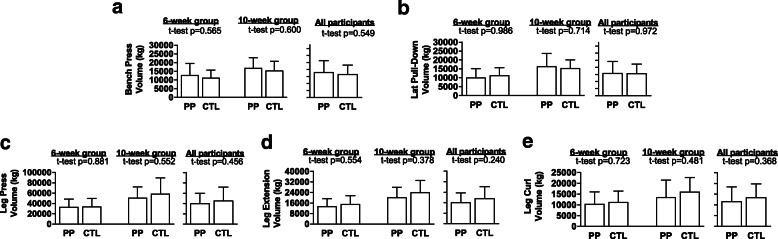
Differences in Exercise Volumes over the Duration of Training. Legend: Data in this figure indicate that bench press volume (panel **a**), lat pulldown volume (panel **b**), leg press volume (panel **c**), leg extension volume (panel **d**), and leg curl volume (panel **e**) did not differ between supplementation groups in the 6-week, 10-week or pooled cohorts. All data are presented as mean ± standard deviation values. Abbreviations: PP, peanut powder supplemented participants; CTL, non-supplemented participants

There were no between-group differences in total volume lifted in the 6-week cohort, 10-week cohort, and pooled cohorts for bench press, cable pull-down, leg press, leg extension or leg curl exercises (Fig. 4a-e).

### Changes in DXA fat mass, LSTM and percent body fat

**Fig. 5:**
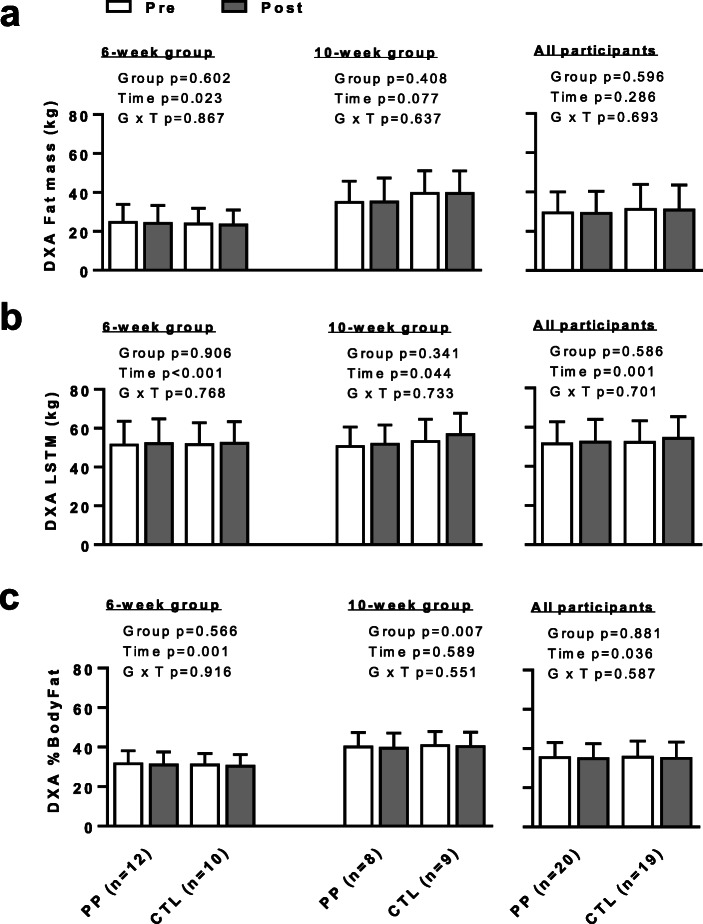
Changes in DXA Fat Mass, LSTM and Percent Body Fat. Legend: Data in this figure indicate that changes in DXA-derived fat mass (panel **a**), DXA-derived lean soft tissue mass (panel **b**), or DXA-derived percent body fat (panel **c**) did not differ between supplementation groups. All data are presented as mean ± standard deviation values. Abbreviations: PP, peanut powder supplemented participants; CTL, non-supplemented participants

There was no significant main effect of group or interaction for fat mass in any of the cohorts. There was, however, a significant main effect of time in the 6-week cohort (*p* = 0.023, Fig. 5a). There was no significant main effect of group or interaction for LSTM in any cohort (Fig. 5b). However, there was a significant main effect of time for LSTM in the 6-week (*p* < 0.001), 10-week (*p* = 0.044) and pooled cohorts (*p* = 0.001). There was no significant GxT interaction for body fat percentage for any cohort (Fig. 5c). However, there was a significant main effect of time in body fat percentage in both the 6-week (*p* = 0.001) and pooled (*p* = 0.036) cohorts, but not the 10-week cohort. Additionally, there was a significant main effect of group for body fat percentage in the 10-week cohort (*p* = 0.007), but not the 6-week or pooled cohorts. Total body mass data are not plotted in Fig. 5. However, there were no significant main effects or GxT interactions for this variable (6-week: group *p* = 0.964, time *p* = 0.059, GxT *p* = 0.912; 10-week: group *p* = 0.070, time *p* = 0.448, GxT *p* = 0.493; pooled cohorts: group *p* = 0.391, time *p* = 0.215, GxT *p* = 0.489).

As an aside, we retroactively screened DXA scans and calculated Appendicular Skeletal Muscle Index (ASMI) for each participant by taking the sum of lean mass in the arms and legs and dividing it by height in meters-squared. Our reasons for doing do were to potentially run sub-analyses on individuals which fell into the sarcopenia range according to the 2010 guidelines put forth by the European Working Group on Sarcopenia in Older People (EWGSOP) (i.e., < 5.86 kg/m^2^ for men and < 4.72 kg/m^2^ in females) [23]. Using these criteria, however, we only discovered that one female in the CTL group presented a value below this threshold (age = 79, ASMI = 4.27). Thus, subanalyses on sarcopenic participants were not performed.

### Changes in mid-thigh VL thickness, mCSA and muscle density

**Fig. 6:**
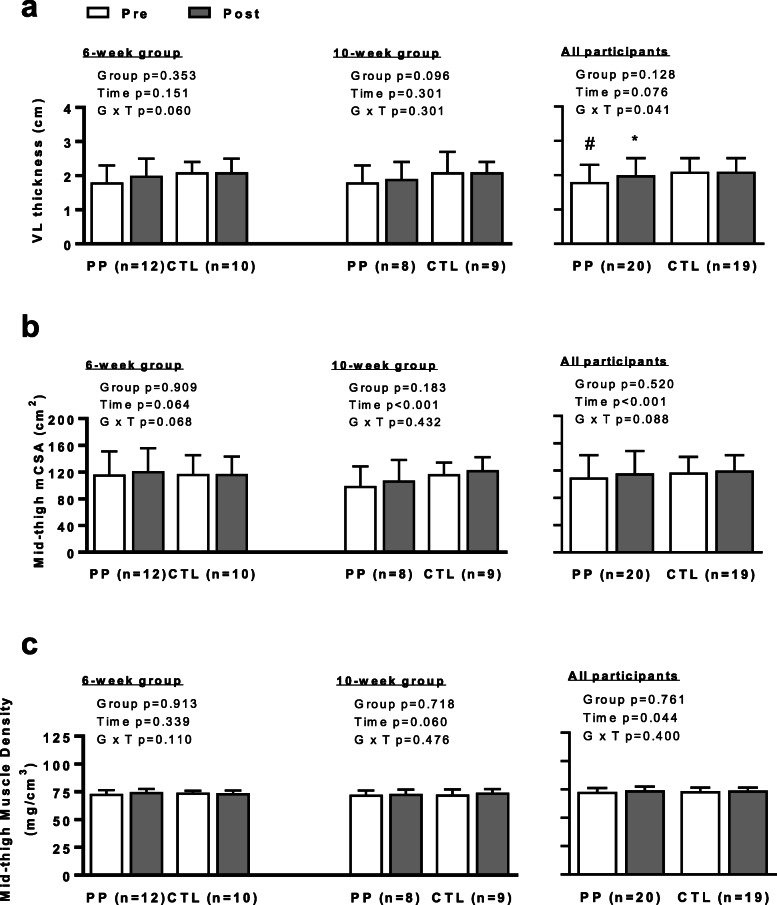
Changes in Mid-thigh Muscle Hypertrophy Measurements. Legend: Data in this figure indicate that vastus lateralis (VL) muscle thickness increased in PP participants when the 6- and 10-week cohorts were pooled, whereas this did not occur in CTL participants (panel **a**). However, a significant interaction was not observed in pQCT-derived mid-thigh lean muscle cross sectional area values (panel **b**) or mid-thigh pQCT-derived muscle density (panel **c**). All data are presented as mean ± standard deviation values. Abbreviations: PP, peanut powder supplemented participants; CTL, non-supplemented participants. Symbols: *, significant increase within PP from Pre to Post (*p* < 0.05); #, PP < CTL at Pre (*p* < 0.05)

There was no GxT interaction for VL thickness in the 6- or 10-week cohorts. However, when pooled, there was a significant GxT interaction for VL thickness (*p* = 0.041). After decomposing the data, post-hoc analyses revealed that VL thickness significantly increased in the PP group (Pre = 1.8 ± 0.4 cm versus Post = 2.0 ± 0.4 cm, *p* < 0.001) (Fig. 6a) but did not change in the CTL (Pre = 2.1 ± 0.5 cm versus Post = 2.1 ± 0.5 cm, *p* > 0.05). Importantly, the change in VL thickness observed in the PP group was greater than the minimal difference to be considered real (0.16 cm). There was no significant GxT interaction for mCSA in any cohort (Fig. 6b). However, it is noteworthy that the interaction effect approached significance in both the 6-week (*p* = 0.068) and pooled cohorts (*p* = 0.088). There was a significant main effect of time for mCSA in the 10-week (*p* = 0.001) and pooled (*p* = 0.001) cohorts, but not the 6-week cohort (*p* = 0.064). Finally, there was no main effect of group for any cohort. After decomposing the mCSA data for the pooled cohort, post-hoc analysis revealed that mid-thigh mCSA significantly increased in the PP group (Pre = 109.6 ± 32.8 cm^2^ versus Post = 115.8 ± 32.8 cm^2^, *p* = 0.01) but not the CTL (Pre = 117.1 ± 22.9 cm^2^ versus Post = 120.0 ± 22.5 cm^2^, *p* = 0.07). There were no significant interaction or main effects for group in the 6-week, 10-week or pooled cohorts for muscle density (Fig. 6c). However, there was a main effect of time for this variable in the pooled cohorts (*p* = 0.044).

### Right leg isokinetic peak torque

**Fig. 7:**
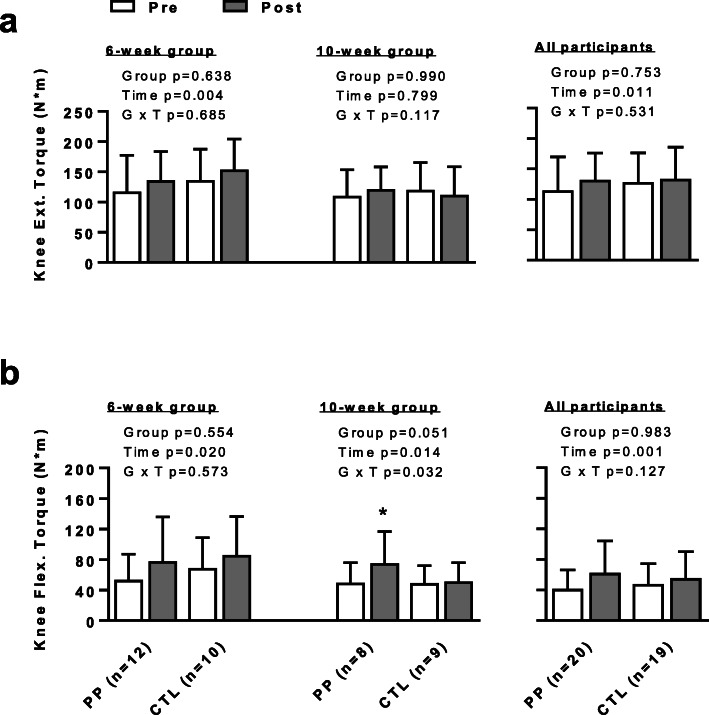
Right Leg Knee Extensor and Flexion Peak Torque. Legend: Data in this figure indicate that knee extensor peak torque increased with training, regardless of supplementation (panel **a**). The same was observed with knee flexion peak torque (panel **b**); however, PP supplementation increased this metric in the 10-week cohort, whereas this metric did not increase in CTL participants. All data are presented as mean ± standard deviation values. Abbreviations: PP, peanut powder supplemented participants; CTL, non-supplemented participants. Symbols: *, significant increase within PP from Pre to Post (*p* < 0.05)

There was no difference in knee extensor torque at 60^0^/s between groups and no GxT interaction. There was, however, a significant effect of time in the 6-week (*p* = 0.004) and pooled (*p* = 0.011) cohorts (Fig. 7a). When testing knee flexor torque at 60^0^/s, there was no difference between groups in any cohort. However, there was a significant effect of time in the 6-week (*p* = 0.020), 10-week (*p* = 0.014) and pooled (*p* = 0.001) cohorts. While there was no interaction in the 6-week and pooled cohorts, there was a significant GxT interaction in the 10-week cohort (*p* = 0.032). The PP group significantly increased knee flexor torque (Pre = 49.5 ± 26.5 N × m versus Post = 75.0 ± 41.9 N × m, *p* = 0.007), whereas the CTL did not (Fig. 7b).

### Additional statistical analyses considering age stratification

Given the wide age spread within each group as discussed above, several analyses were performed. First, Pearson correlations were performed between age and baseline values for key dependent variables in all 39 participants (i.e., DXA LSTM, ultrasound-derived VL thickness, pQCT-derived mCSA and muscle density, and knee extensor/flexion metrics). Results from these analyses were as follows: age versus DXA LTSM *r* = − 0.152, *p* = 0.354; age versus VL thickness *r* = − 0.347, *p* = 0.036; age versus pQCT-derived mCSA *r* = − 0.193, *p* = 0.240; age versus pQCT-derived muscle density *r* = − 0.325, *p* = 0.044; age versus knee extensor strength *r* = − 0.433, *p* = 0.006; age versus knee flexion strength *r* = − 0.288, *p* = 0.075. We interpret these results to suggest certain metrics prior to training may have been adversely impacted by age; specifically, VL thickness, muscle density, and knee extensor strength.

Next, Pearson correlations were performed between age and POST-PRE (i.e., “delta”) scores for all of the aforementioned dependent variables in all 39 participants. Interestingly, only the PRE-to-POST change in knee extension strength was significantly associated with age (*r* = 0.595, *p* < 0.0001), and this was a positive association suggesting older individuals gained more knee extensor strength, regardless of supplementation. All other delta scores showed no significant associations with age (delta DXA LSTM *r* = − 0.272, *p* = 0.094; delta knee flexion strength *r* = 0.045, *p* = 0.785; delta VL thickness *r* = 0.014, *p* = 0.933; delta pQCT mCSA *r* = − 0.150, *r* = 0.363; delta pQCT muscle density *r* = 0.229, *r* = 0.162). We interpret these results to suggest that these key training adaptations were minimally influenced by age, regardless of supplementation.

**Fig. 8:**
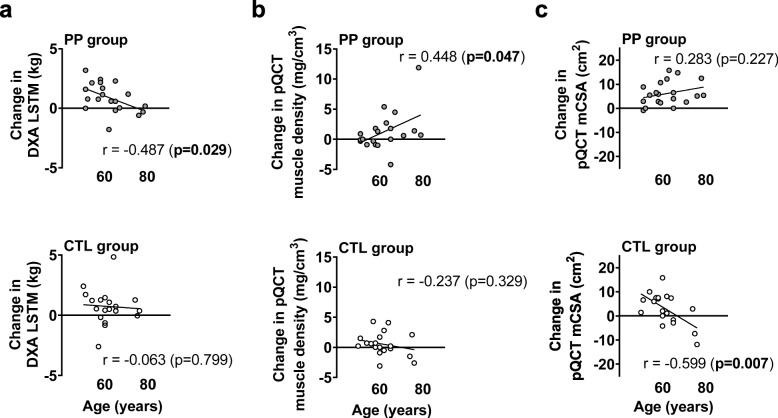
Select correlations between age and key dependent variables in each supplement group. Legend: These data show correlations between age and change in DXA LSTM (panel **a**), change in pQCT muscle density (panel **b**), and change in pQCT mCSA (panel **c**). Abbreviations: PP, peanut powder supplemented participants; CTL, non-supplemented participants

Finally, we performed Pearson correlations within each group to assess if age was associated with the aforementioned delta scores with and without PP supplementation. Key findings are presented in Fig. 8. Within the PP group, age was negatively associated with delta DXA LSTM (*r* = − 0.487, *p* = 0.029, Fig. 8a), and this relationship was not significant in the CTL group (*r* = − 0.063, *p* = 0.799). Conversely, within the PP group, age was positively associated with delta pQCT muscle density (*r* = 0.448, *p* = 0.049, Fig. 8b), and this relationship was not significant in the CTL group (*r* = − 0.273, *p* = 0.329). Within the PP group, age was not associated with delta pQCT mCSA (*r* = 0.283, *p* = 0.227, Fig. 8c), whereas age was negatively associated with this variable in the CTL group (*r* = − 0.599, *p* = 0.007). No between-group divergences occurred when within-group correlations were performed for age versus knee extensor/flexion strength or VL thickness (data not shown). The potential significance of these findings will be discussed below.

### Additional statistical analyses in the PP group when considering gender

We were also interested in determining if change scores in key training adaptations differed between genders in the PP group (*n* = 8 females, *n* = 12 males). In short, change scores between PP males and females were as follows: change in DXA LSTM, females = 0.84 ± 1.66 kg, males = 0.58 ± 0.88 kg, *p* = 0.872; change in VL thickness, females = 0.14 ± 0.14 cm, males = 0.16 ± 0.15 cm, *p* = 0.499; change in pQCT mCSA, females = 5.73 ± 3.71 cm^2^, males = 7.48 ± 6.49 cm^2^, *p* = 0.747; change in pQCT muscle density, females = 0.64 ± 2.69 mg/cm^3^, males = 2.29 ± 4.33 mg/cm^3^, *p* = 0.493; change in knee extension peak torque, females = 12.0 ± 18.3 N × m, males = 20.6 ± 25.1 N × m, *p* = 0.480; change in knee flexion peak torque, females = 16.8 ± 17.4 N × m, males = 24.3 ± 16.1 N × m, *p* = 0.550. Collectively, these data suggest that gender did not play a role in training adaptations in the PP group.

## Discussion

There is good evidence to suggest that protein supplementation with RT enhances various training adaptations (reviewed in [24]). Notwithstanding, the majority of these studies have examined the effects of animal-based or soy protein supplements, and no studies to date have evaluated the efficacy of RT with PP supplementation on measures of muscle mass, function and strength.

There are several noteworthy findings herein. First, PP supplementation significantly increased knee flexion peak torque in the 10-week cohort relative to the CTL group. Additionally, when the 6- and 10-week cohorts were pooled, PP participants experienced significant increases in VL thickness compared to CTL participants. A similar trend was also observed regarding mid-thigh mCSA; specifically, the interaction trended (*p* = 0.088) and forced post hoc tests indicated that this metric increased in the PP participants from pre- to post-training (*p* < 0.05), whereas there was not a significant change in CTL participants. Our dynamometry data align with various studies that have reported RT with protein supplementation enhances lower-body leg strength relative to placebo supplementation [25, 26]. Likewise, our muscle imaging data agree with various studies demonstrating protein supplementation with RT enhances muscle mass relative to placebo supplementation [26–29]. However, there are also data showing that protein supplementation with RT does not affect variables related to muscle hypertrophy or strength in older individuals [30–32]. Discrepancies between studies are likely due to various factors including the type of protein administered as well as the duration and type of RT. There is strong evidence to suggest protein needs (specifically, the intake of more essential amino acids) increase with age due to decreases in gastrointestinal function and anabolic resistance in skeletal muscle [33, 34]. As mentioned prior, protein isolated from peanuts contains a full complement of amino acids. It is also notable that several of the studies cited above have examined the effects of whey protein with RT, and positive findings from these studies are likely due to the high leucine and essential amino acid content as well as the high protein digestibility corrected amino acid score of whey [35]. Based on data from Table 1, the daily serving of PP provided roughly 30 g of protein, 9.2 g of essential acids (note, tryptophan was not assessed or included), and 2.2 g leucine. In comparison, 30 g of a whey protein isolate provides roughly 15 g of essential amino acids and ~ 3.5 g of leucine [35]. Thus, whether the training adaptations observed herein were due to the PP participants consuming more calories, and/or whether supplementation with a product containing a high amount of peanut protein would be equally as good as or less effective in enhancing training adaptations relative to whey protein supplementation remains to be determined. Finally, while the current findings with PP supplementation are promising, we posit that they are preliminary in nature given that the increase in VL thickness in the pooled PP subjects, while significant compared to the CTL subjects, was relatively small (+ 0.20 cm, or an 11% increase on average) and only modestly above the minimal difference precision value for ultrasound imaging (+ 0.16 cm). Thus, future studies that are longer in duration are needed to definitively determine if PP supplementation can enhance hypertrophic adaptations with resistance training.

Contrary to the data above, PP supplementation after one bout of resistance exercise did not enhance integrated MyoPS rates within a 24 h period following the first training bout. This finding is difficult to reconcile given that PP supplementation enhanced various training adaptations as described above. However, it is notable that post-exercise increases in MyoPS rates hours following an exercise bout have been shown to demonstrate poor agreement with long-term hypertrophic outcomes (reviewed in [36]). RT studies examining integrated MyoPS rates using D_2_O over days or weeks into training have yielded better associations with hypertrophic outcomes [21, 37, 38]. However, again, these correlations are modest at best. Therefore, we posit that the current MyoPS data continue to suggest that tracer data should be viewed independently of chronic training outcomes. Moreover, had we used an acute tracer infusion protocol with a phenylalanine stable isotope, we may have observed enhanced post-exercise MyoPS rates compared to no supplementation within a more acute time frame (i.e., 3–6 h). Thus, given the paucity of data in this area, future research is needed to examine how the ingestion of different PP doses acutely affect MyoPS rates relative to other protein supplements.

A unique aspect of this study is the implementation of the pQCT to ascertain muscle quality. The pQCT provides muscle quality through the assessment of muscle density [39, 40]. An age-associated “drift” in muscle density can be due to either: a) an increased infiltration of fat tissue, and/or b) a decreased proportion of muscle protein. While no interactions existed for the metrics provided, it is interesting that muscle density increased over the duration of training, regardless of supplementation. This metric is not commonly reported in the exercise physiology literature given that pQCT and CT scanners are not readily available. However, our data agree with a study by Claassen et al. [41] where the authors used a CT scanner to report that 6 weeks of RT increased Hounsfield units of the mid-thigh by ~ 4–5% in college-aged men. In explaining their findings, the authors speculated that the observed increase in muscle density was due to either an increase in connective tissue density and/or an increase in contractile protein density. Thus, we interpret our data to suggest that RT increases muscle density through an increase in contractile and/or connective tissue density. Notably, this is an important finding given that a higher muscle density has been shown to be associated with physical function in overweight/obese older participants [42].

While preliminary, our subanalyses on age and gender provide insightful information. In short, the lack of differences in change scores for key training adaptations between males and females in the PP group suggests gender does not affect responses to supplementation. In agreement with our gender analyses, other studies have shown that and females exhibit similar strength and hypertrophic responses to resistance training [43], as well as protein supplementation [24]. Nevertheless, future studies that are more well-powered are needed to determine if gender plays an appreciable role in the responses to PP supplementation. Contrary to the gender analysis, our subanalysis on age effects (Fig. 8 data) indicate that some age-dependent adaptations may have occurred within each supplementation group. First, the negative correlation between age and change in DXA LSTM in the PP group may suggest that PP supplementation could be more beneficial in individuals less than ~ 60 years old, and relatively ineffective in individuals that are older. However, this interpretation is confounded by older CTL participants demonstrating hypertrophy across the age spectrum. Further confounding this interpretation are the pQCT data, which show that mCSA seemingly increased in an age-dependent fashion in PP participants. Our two-way statistics indicated that VL thickness and mCSA increased in PP versus CTL participants. Thus, if age does play a small role in PP-induced skeletal muscle hypertrophy with resistance training, we speculate that older individuals may experience more of a benefit. However, given our limited n-sizes, more data are needed to validate this hypothesis.

What should finally be noted is the lack of agreement between some of our body composition metrics. Although PP supplementation was found to increase vastus lateralis hypertrophy, no interactions between supplement groups were observed for whole-body DXA LSTM. Additionally, although a significant interaction was not observed for pQCT-derived mid-thigh mCSA changes, the interaction trended (*p* = 0.088) as discussed above. While these data are difficult to reconcile, we have noted in the past that the agreement between methods used to assess skeletal muscle hypertrophy poorly agree with one another [44]. Thus, our data re-iterate the notion that various measures of muscle mass determination do not exhibit good agreement, and these findings continue to warrant future research in this area.

### Limitations

A notable limitation of the current study is the duration of the intervention of the second cohort. Given the unforeseen consequences of the SARS-CoV-2 pandemic, we voluntarily decided to prematurely end the second cohort 4 weeks early rather than jeopardize the health and safety of our participants. Another unresolved limitation was that, while most participants reported to the laboratory in the morning for his/her testing batteries, 7 participants had to report in the evening after shorter fasts due to scheduling conflicts. In spite of this logistical constraint, we ensured that these 7 participants returned to the laboratory for post-testing procedures during the same evening times. These data are also limited given the large spread in age in each group, albeit our correlation subanalyses suggested that age (regardless of supplementation) likely did not affect training adaptations. While males and females completed the intervention, a lack of statistical power precluded a more comprehensive determination of gender interactions with PP supplementation. Nonetheless, our preliminary gender analyses suggest gender does not influence training adaptations in the PP group. What should finally be noted is that we lack segmental extracellular fluid and intracellular fluid data from the legs. We attempted to safeguard from the potential counfounder of edema by having participants report to the laboratory 72 h following their last bout of training. However, it is still possible that changes lower limb ultrasound and pQCT metrics may have been due a combination of fluid shifts and the accretion of muscle proteins as we have posited in the past [44].

## Conclusions

While preliminary, the results of the current study indicate that PP supplementation with 6–10 weeks of RT enhance certain aspects of muscle hypertrophy and strength in older adults, compared to a RT program alone in the elderly population.

## Data Availability

All raw data can be obtained by emailing the corresponding author (mdr0024@auburn.edu).

## Abbreviations

CTL: Control
DXA: Dual-energy x-ray absorptiometry
FM: Fat mass
GxT: Group-by-time interaction
LSTM: Lean soft tissue mass
mCSA: Muscle cross sectional area
MyoPS: Myofibrillar protein synthesis
PP: Peanut protein
pQCT: Peripheral quantitative computed tomography
RT: Resistance training
VL: Vastus lateralis

## References

[1] Deschenes MR (2004). Effects of aging on muscle fibre type and size. Sports Med.

[2] Lexell J. Human aging, muscle mass, and fiber type composition. J Gerontol A Biol Sci Med Sci. 1995;50 Spec No:11–6. 10.1093/gerona/50a.special_issue.11. 10.1093/gerona/50a.special_issue.11 7493202

[3] Rosenberg IH (1989). Summary comments. Am J Clin Nutr.

[4] Mankhong S, Kim S, Moon S, Kwak HB, Park DH, Kang JH. Experimental Models of Sarcopenia: Bridging Molecular Mechanism and Therapeutic Strategy. Cells. 2020;9(6):1385. 10.3390/cells9061385 PMC7348939 32498474

[5] Siparsky PN, Kirkendall DT, Garrett WE (2014). Muscle changes in aging: understanding sarcopenia. Sports Health.

[6] Volpi E, Nazemi R, Fujita S (2004). Muscle tissue changes with aging. Curr Opin Clin Nutr Metab Care..

[7] Beaudart C, Zaaria M, Pasleau F, Reginster JY, Bruyere O (2017). Health outcomes of sarcopenia: a systematic review and meta-analysis. PLoS One.

[8] Hunter GR, McCarthy JP, Bamman MM (2004). Effects of resistance training on older adults. Sports Med.

[9] Fragala MS, Cadore EL, Dorgo S, Izquierdo M, Kraemer WJ, Peterson MD (2019). Resistance training for older adults: position statement from the national strength and conditioning association. J Strength Cond Res.

[10] Tang JE, Phillips SM (2009). Maximizing muscle protein anabolism: the role of protein quality. Curr Opin Clin Nutr Metab Care.

[11] Burd NA, Beals JW, Martinez IG, Salvador AF, Skinner SK (2019). Food-first approach to enhance the regulation of post-exercise skeletal muscle protein synthesis and remodeling. Sports Med.

[12] Phillips SM (2016). The impact of protein quality on the promotion of resistance exercise-induced changes in muscle mass. Nutr Metab (Lond).

[13] Nelson ME, Hamm MW, Hu FB, Abrams SA, Griffin TS (2016). Alignment of healthy dietary patterns and environmental sustainability: a systematic review. Adv Nutr.

[14] Wang DD, Leung CW, Li Y, Ding EL, Chiuve SE, Hu FB (2014). Trends in dietary quality among adults in the United States, 1999 through 2010. JAMA Intern Med.

[15] de Boer J, Aiking H (2018). Prospects for pro-environmental protein consumption in Europe: cultural, culinary, economic and psychological factors. Appetite..

[16] Arya SS, Salve AR, Chauhan S (2016). Peanuts as functional food: a review. J Food Sci Technol.

[17] Singh B, Singh U (1991). Peanut as a source of protein for human foods. Plant Foods Hum Nutr.

[18] Haun CT, Mumford PW, Roberson PA, Romero MA, Mobley CB, Kephart WC, et al. Molecular, neuromuscular, and recovery responses to light versus heavy resistance exercise in young men. Physiol Rep. 2017;5(18):e13457. 10.14814/phy2.13457. 10.14814/phy2.13457 PMC5617935 28963127

[19] Evans WJ, Phinney SD, Young VR (1982). Suction applied to a muscle biopsy maximizes sample size. Med Sci Sports Exerc.

[20] Roberts MD, Young KC, Fox CD, Vann CG, Roberson PA, Osburn SC (2020). An optimized procedure for isolation of rodent and human skeletal muscle sarcoplasmic and myofibrillar proteins. J Biol Methods.

[21] Bell KE, Brook MS, Snijders T, Kumbhare D, Parise G, Smith K (2019). Integrated myofibrillar protein synthesis in recovery from unaccustomed and accustomed resistance exercise with and without multi-ingredient supplementation in overweight older men. Front Nutr.

[22] Subar AF, Kirkpatrick SI, Mittl B, Zimmerman TP, Thompson FE, Bingley C (2012). The automated self-administered 24-hour dietary recall (ASA24): a resource for researchers, clinicians, and educators from the National Cancer Institute. J Acad Nutr Diet.

[23] Cruz-Jentoft AJ, Baeyens JP, Bauer JM, Boirie Y, Cederholm T, Landi F (2010). Sarcopenia: European consensus on definition and diagnosis: report of the European working group on sarcopenia in older people. Age Ageing.

[24] Morton RW, Murphy KT, McKellar SR, Schoenfeld BJ, Henselmans M, Helms E (2018). A systematic review, meta-analysis and meta-regression of the effect of protein supplementation on resistance training-induced gains in muscle mass and strength in healthy adults. Br J Sports Med.

[25] Gryson C, Ratel S, Rance M, Penando S, Bonhomme C, Le Ruyet P (2014). Four-month course of soluble milk proteins interacts with exercise to improve muscle strength and delay fatigue in elderly participants. J Am Med Dir Assoc.

[26] Chale A, Cloutier GJ, Hau C, Phillips EM, Dallal GE, Fielding RA (2013). Efficacy of whey protein supplementation on resistance exercise-induced changes in lean mass, muscle strength, and physical function in mobility-limited older adults. J Gerontol A Biol Sci Med Sci.

[27] Tieland M, Dirks ML, van der Zwaluw N, Verdijk LB, van de Rest O, de Groot LC (2012). Protein supplementation increases muscle mass gain during prolonged resistance-type exercise training in frail elderly people: a randomized, double-blind, placebo-controlled trial. J Am Med Dir Assoc.

[28] Dirks ML, Tieland M, Verdijk LB, Losen M, Nilwik R, Mensink M (2017). Protein supplementation augments muscle Fiber hypertrophy but does not modulate satellite cell content during prolonged resistance-type exercise training in frail elderly. J Am Med Dir Assoc.

[29] Verdijk LB, Jonkers RA, Gleeson BG, Beelen M, Meijer K, Savelberg HH (2009). Protein supplementation before and after exercise does not further augment skeletal muscle hypertrophy after resistance training in elderly men. Am J Clin Nutr.

[30] Bjorkman MP, Pilvi TK, Kekkonen RA, Korpela R, Tilvis RS (2011). Similar effects of leucine rich and regular dairy products on muscle mass and functions of older polymyalgia rheumatica patients: a randomized crossover trial. J Nutr Health Aging.

[31] Leenders M, Verdijk LB, Van der Hoeven L, Van Kranenburg J, Nilwik R, Wodzig WK (2013). Protein supplementation during resistance-type exercise training in the elderly. Med Sci Sports Exerc.

[32] Candow DG, Chilibeck PD, Facci M, Abeysekara S, Zello GA (2006). Protein supplementation before and after resistance training in older men. Eur J Appl Physiol.

[33] Baum JI, Kim IY, Wolfe RR. Protein consumption and the elderly: What is the optimal level of intake? Nutrients. 2016;8(6):359. 10.3390/nu8060359. 10.3390/nu8060359 PMC4924200 27338461

[34] Burd NA, McKenna CF, Salvador AF, Paulussen KJM, Moore DR (2019). Dietary protein quantity, quality, and exercise are key to healthy living: a muscle-centric perspective across the lifespan. Front Nutr..

[35] Hulmi JJ, Lockwood CM, Stout JR (2010). Effect of protein/essential amino acids and resistance training on skeletal muscle hypertrophy: A case for whey protein. Nutr Metab (Lond).

[36] Mitchell CJ, Churchward-Venne TA, Cameron-Smith D, Phillips SM (2015). What is the relationship between the acute muscle protein synthesis response and changes in muscle mass?. J Appl Physiol (Bethesda, Md : 1985).

[37] Brook MS, Wilkinson DJ, Mitchell WK, Lund JN, Szewczyk NJ, Greenhaff PL (2015). Skeletal muscle hypertrophy adaptations predominate in the early stages of resistance exercise training, matching deuterium oxide-derived measures of muscle protein synthesis and mechanistic target of rapamycin complex 1 signaling. FASEB J.

[38] Damas F, Phillips SM, Libardi CA, Vechin FC, Lixandrao ME, Jannig PR (2016). Resistance training-induced changes in integrated myofibrillar protein synthesis are related to hypertrophy only after attenuation of muscle damage. J Physiol.

[39] Sherk VD, Thiebaud RS, Chen Z, Karabulut M, Kim SJ, Bemben DA (2014). Associations between pQCT-based fat and muscle area and density and DXA-based total and leg soft tissue mass in healthy women and men. J Musculoskelet Neuronal Interact.

[40] Goodpaster BH, Kelley DE, Thaete FL, He J, Ross R (2000). Skeletal muscle attenuation determined by computed tomography is associated with skeletal muscle lipid content. J Appl Physiol (Bethesda, Md : 1985).

[41] Claassen H, Gerber C, Hoppeler H, Lüthi JM, Vock P (1989). Muscle filament spacing and short-term heavy-resistance exercise in humans. J Physiol.

[42] Scott D, Shore-Lorenti C, McMillan LB, Mesinovic J, Clark RA, Hayes A (2018). Calf muscle density is independently associated with physical function in overweight and obese older adults. J Musculoskelet Neuronal Interact.

[43] Roberts BM, Nuckols G, Krieger JW (2020). Sex differences in resistance training: a systematic review and meta-analysis. J Strength Cond Res..

[44] Haun CT, Vann CG, Roberts BM, Vigotsky AD, Schoenfeld BJ, Roberts MD (2019). A critical evaluation of the biological construct skeletal muscle hypertrophy: size matters but so does the measurement. Front Physiol.

